# The Treatment of Sea-Sickness

**Published:** 1920-03-20

**Authors:** 


### THE TREATMENT OF SEA-SICKNESS.

### A New Method and its Theory.

THE means by which the sufferings of the landsman afloat can be relieved form a subject which has received, for ages past, so much professional and quack attention that it seems almost impossible that a new and simple treatment upon rational lines could be invented.

A short time ago, however, certain writers of the French school put forward and, indeed, claim success for, the atropine-adrenalin method. The resumption on a large scale of ocean passenger traffic, the likelihood of an increasing number of people being faced with the problem of keeping reasonable comfort of body during many weeks at sea, and correspondingly increasing their demands for medical advice upon the subject, make it worth while at least considering the possibilities of the new suggestions.

We may confine ourselves here to a brief outline of the principle, and as the actual treatment is absolutely simple in technique, the result must depend largely upon the judgment with which the drugs and their exact doses are administered. The point made is that sea-sickness must be restudied from the standards set up for endocrinology and the pathology of the sympathetic nervous system. It is stated that during the typical state of nausea, headache, etc., the arterial tension is progressively increased up to a certain point and then falls steadily as the malaise continues. In an attempt to attribute the changes to causes other than nervous abnormalities a series of lumbar punctures and blood counts were performed, but no consistent results were obtained and nothing to suggest that the pathology would be elucidated by such means.

Clinically, however, there were definite signs of a state of hyper-excitability of the sympathetic system recorded by one set of observers, while, on the other hand, another set found exactly the reverse condition, namely, a vagotonia, to be present. More recently the two views are reconciled by the announcement that, at the onset of symptoms, first the sympathetic system is at an abnormally high tone and later, as the days of illness continue, this falls and is finally reversed, the vagus system then having the upper hand in the balance. If we care to theorise on the point, it may be that the initial sympathetic or adrenal activity causes a gradual exhaustion of the cells in the adrenal cortex, and so, after a short time, vagus effects and predominance come more and more to fill the picture. In any case, the French observations would seem to suggest that sea-sickness has its pathology based upon the relative activity of the adrenal cells, and therefore upon the tone of the sympathetic system.

Thus we come to the interesting question of which persons are and which are not predisposed to *mal de mer*. It will be generally agreed that those who exhibited a highly-strung, sympathetic system are generally the more susceptible, while the converse is also true. A case in point is the relative immunity enjoyed by the very young child, in whom the vagus system is invariably the more important for the time being. Also, true vomiting crises—vagotonic paroxysms—are more frequently met with in protracted cases of illness where the gradual exhaustion of the adrenal element has previously occurred.

So much for the theory, and, if we view this as a possible pathology for the condition, then treatment along certain lines is an obvious deduction. If the sympathetic tone is up, administration of adrenalin would obviously aggravate it; but if it has fallen, as it will have in the majority of severe cases coming under the doctor's notice, then adrenalin will act as a valuable antidote to the vagus predominance. Otherwise, for severe vomiting and other critical vagus manifestations we can use the simple atropine injections.

Thus the first point to be decided is apparently the stage of the process encountered, and although there does not at first sight seem to be more in this treatment than a theory and a rational control for the later stages of acute sea-sickness, yet the whole matter is viewed in an entirely new and simple light, and under those conditions is well worthy of passing attention.

